# Two variants of uterine leiomyoma in Malaysia’s last Sumatran rhinoceros (*Dicerorhinus sumatrensis*)

**DOI:** 10.1080/01652176.2020.1836431

**Published:** 2020-10-22

**Authors:** Annas Salleh, Zainal Zahari Zainuddin, Mohamed Reza Mohamed Tarmizi, Keng Chee Yap, Mohd Zamri-Saad

**Affiliations:** a Faculty of Veterinary Medicine, Department of Veterinary Laboratory Diagnosis, Universiti Putra Malaysia, Serdang, Malaysia; b Borneo Rhino Alliance, Sabah, Malaysia

**Keywords:** Sumatran rhinoceros, *Dicerorhinus sumatrensis*, reproductive tract, pathology, leiomyoma, immunohistochemistry

## Abstract

Following its capture in March 2014, an adult female Sumatran rhinoceros frequently showed profuse vaginal bleeding. An ultrasonography suggested the presence of multiple reproductive lesions, including two uterine masses which were suspected to be leiomyomas. Soon after, an open pyometra was confirmed. Later in November 2019, the patient died and necropsy confirmed the presence of two uterine masses; one was located at the cervico-uterine junction and another in the uterine body, with pyometra, and cystic endometrial hyerplasia. Based on histological, special stains, and immunohistochemical examination, it was shown that one of the masses was composed of large, ovoid and polyhedral neoplastic mesenchymal cells with eosinophilic cytoplasm and a few binucleated cells surrounded by collagen fibres. It was tested positive for SMA and vimentin, while negative for desmin, cytokeratin AE1/AE3, EMA, CD34, and S100. The other mass was composed of mesenchymal cells undergoing myxoid degeneration as evidenced by the presence of glycosaminoglycan-rich matrix. It was tested positive for SMA, vimentin, partially positive for desmin, and negative for the other markers. With the aid of human medical nomenclature, these masses were diagnosed as epithelioid leiomyoma and myxoid leiomyoma, respectively. This report provides a clinical presentation, and histologic descriptions of the two variants of leiomyomas that have not been reported in veterinary medicine.

A female Sumatran rhinoceros (*Dicerorhinus sumatrensis*), estimated to be between 20 and 25 years old (Kitchener 1997; Hillman-Smith et al. 1986) was captured in Danum Valley, Sabah, Malaysia in 2014 and placed in a temporary boma within the forest. The following day, frank blood was observed flowing from its vagina, each time the rhinoceros was laying down. The animal was eventually translocated to the captive facility at Tabin Wildlife Reserve, Sabah eight days later. Discharge from the vagina was observed almost every day in various forms; haemorrhagic, mucoid, suppurative, or mixed, where oral hematinics (Sangobion®, Merck, Petaling Jaya, Malaysia) and antibiotics were administered. Intravenous fluid therapy and antibiotics were delivered via the marginal ear vein only when it had profuse vaginal bleeding (Figure 1) and appeared weak. Intravenous treatment was required 1–2 times a year. Subsequent transrectal ultrasonography confirmed multiple reproductive tract lesions, including endometrial cysts, hydrometra, hydrosalpinx, and two uterine masses. The endometrial cysts were observed as multiple round anechoic, thin-walled structures with smooth margin and of variable sizes ranging from 0.2 cm to 2.0 cm. They were randomly distributed within the uterus that had a heterogenous echotexture (Figure 2a). Hydrometra was observed as thin hyperechoiec uterine wall filled with fluid with mildly echogenic gravity dependent material (Figure 2b). One uterine mass was large, about 15 cm in diameter, and visible as echogenic structure of soft tissue with heterogenous echotexture, filling the uterine lumen (Figure 2c). Another uterine mass was smaller, 4 cm in diameter, with heterogenous echogenic centre and grainy hyperechoic periphery, which also filled the uterine lumen (Figure 2d).

**Figure 1.**
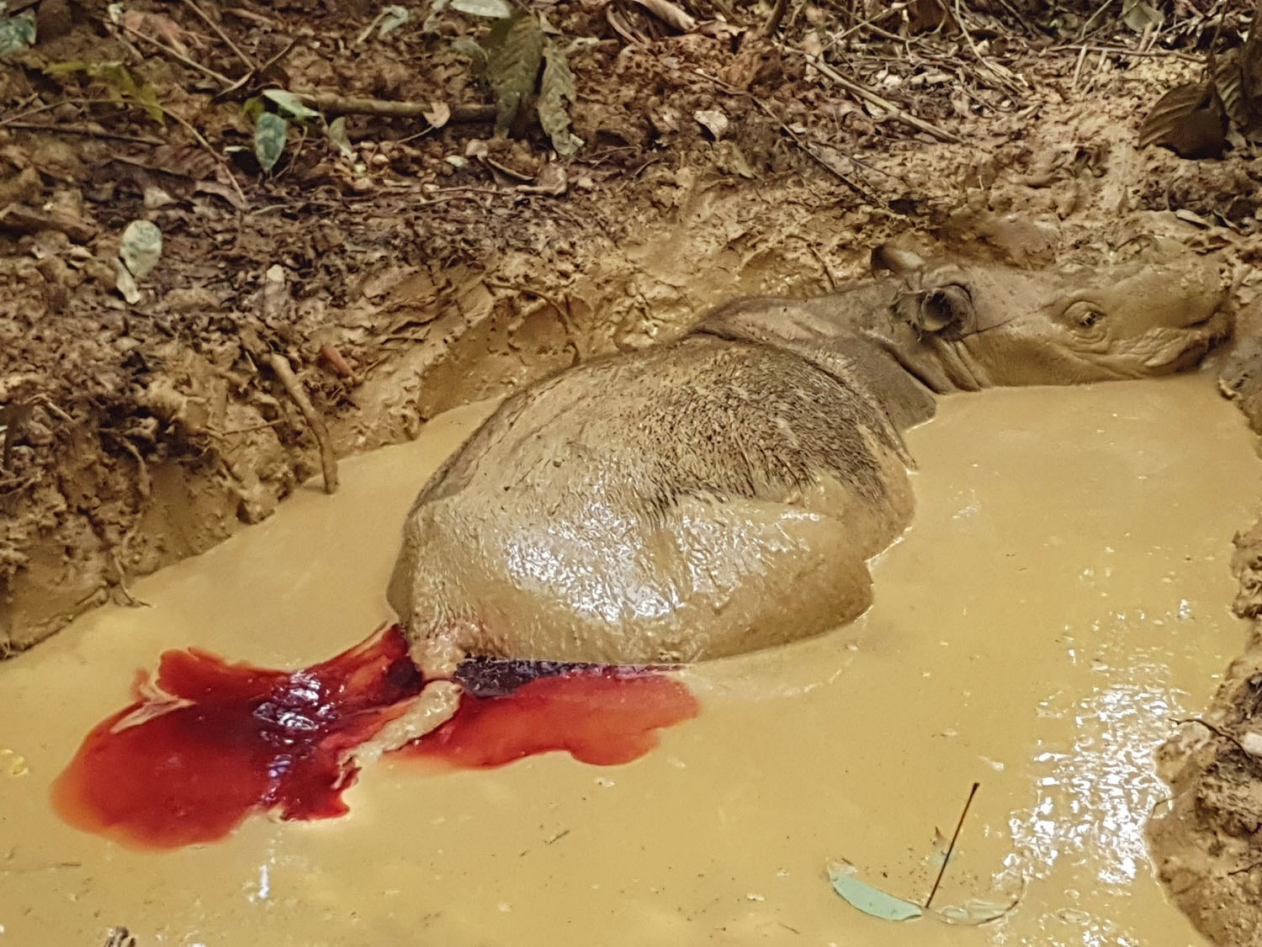
Profuse vaginal bleeding was occasionally observed in a female Sumatran rhinoceros estimated to be between 20 and 25 years old.

**Figure 2.**
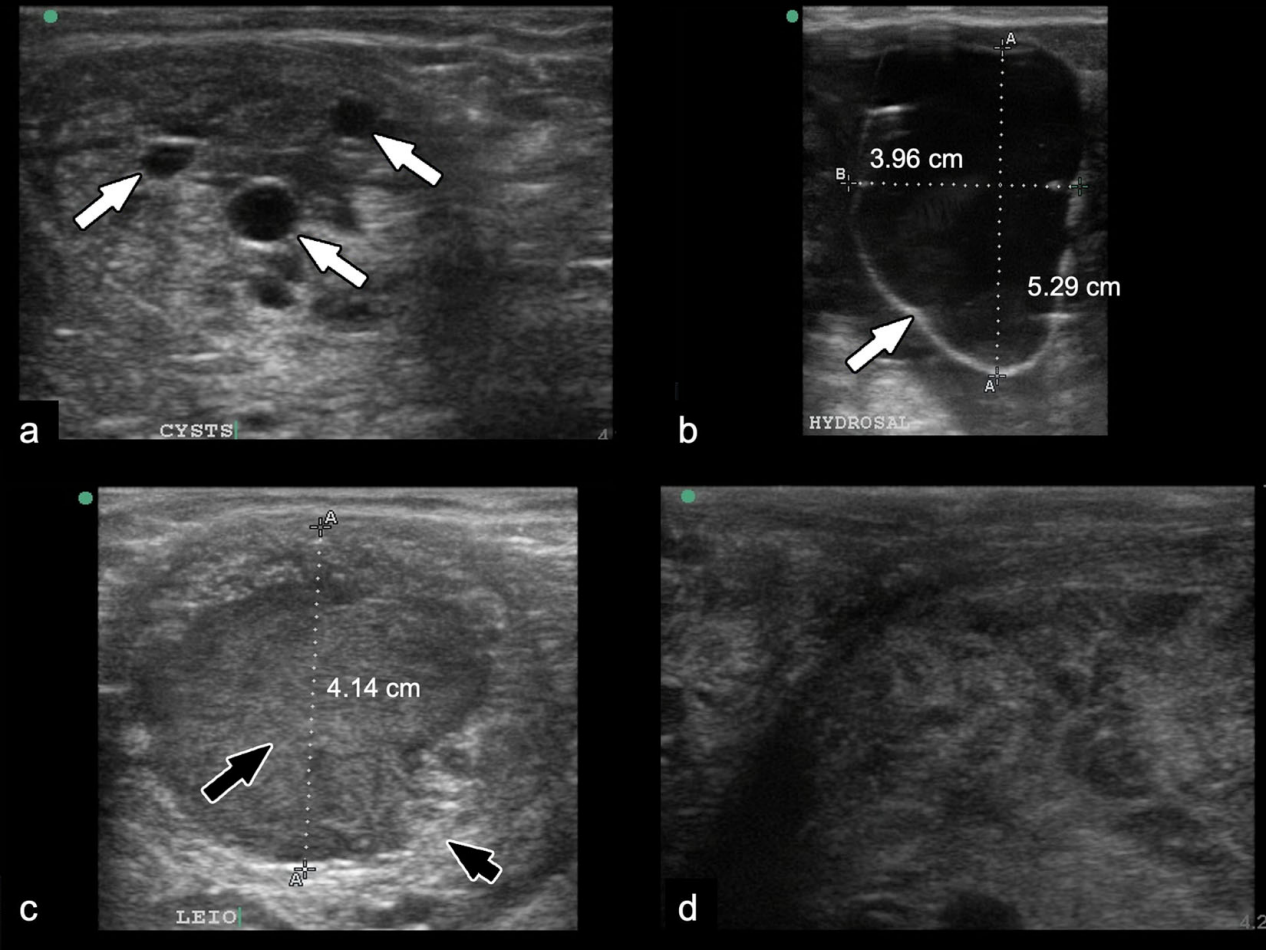
Transrectal ultrasonography of the reproductive tract of a female Sumatran rhinoceros estimated to be between 20 and 25 years old. (a) Multiple round anechoic structures of variable sizes (arrows) within the endometrium suggestive of cystic endometriosis. (b) Hyperechoiec uterine wall (arrow) filled with fluid with mildly echogenic gravity dependent materials. (c) The small uterine mass showing heterogenous echogenic center (long arrow) and grainy hyperechoic periphery (short arrow). (d) The large uterine mass composed of soft tissue echogenic structure with heterogenous echotexture.

Differential diagnoses for the uterine masses included leiomyoma, adenoma, carcinoma, and carcinosarcoma. Surgical removal of the uterine masses was not attempted due to the high risk of post-surgical death caused by bleeding (Foose 1999; Klein et al. 1997). Open pyometra was evident through occasional mucopurulent vaginal discharge. Alpha and beta hemolytic *Streptococcus* sp. were later isolated from the vaginal swabs. Treatments were attempted via uterine flushing, transrectal aspirations of fluids from cysts and hydrosalpinx, and administration of gonadotropin-releasing hormone (Improvac®, Zoetis, Petaling Jaya, Malaysia), however, all were ineffective. Despite these lesions, the rhinoceros still came into oestrus every month. In January 2019, the uterine mass was palpable via the rectum and partially obstructing the rectum. It also prevented transrectal ultrasound imaging of the right ovary, therefore, the rhinoceros showed signs of discomfort during the transrectal ultrasound procedure. Between January and November 2019, it was noticed that the daily browse intake had gradually reduced from 25.5 kg in January to 17.4 kg in November. Its body weight also decreased from 532 kg in January 2019 to 465 kg in November 2019 (Figure 3). In November 2019, pain and discomfort were noted, especially during urination and defecation. It only voided a small amount of urine and faeces each time, and often showed trembling of the hind limbs. During defecation, the rectal straining was associated with the expulsion of blood, mucus, and sometimes small pieces of endometrium through the vagina. Other sign of pain was loud abnormal bleating, which progressed to gaping of the mouth, salivation, and teeth grinding. Prior to death on 23^rd^ November 2019, 3 litres of intravenous infusion consisting of 5% Dextrose, NaCl, and Lymelite® (Vimedin Corporation, Can Tho, Vietnam) along with 22 ml of flunixine meglumine were administered. It was in sternal recumbency before it finally collapsed and died.

**Figure 3.**
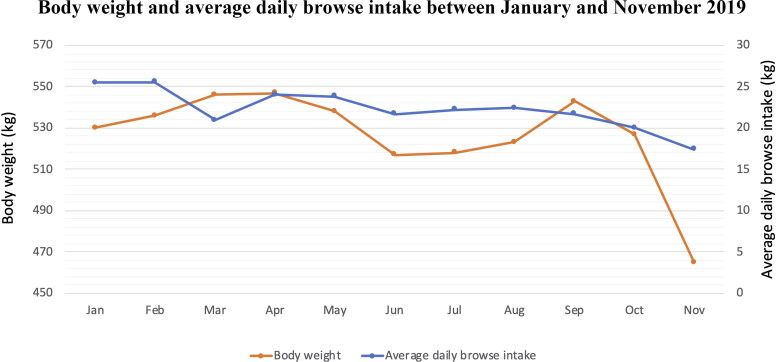
Body weight and average daily browse intake of the rhinoceros between January and November 2019.

The necropsy revealed a generally dehydrated and emaciated carcass, with obvious skin folds. Generalised muscular atrophy was prominent with minimal fat deposits, leading to visible bony protuberances at the pelvis, scapulae and vertebrae. The lungs were also congested, while generalised pale areas were observed at the epicardium and myocardium. The liver was observed with degeneration and necrosis in all lobes. Mild haemorrhages of the serosa were observed at the caeco-colic junction. Kidneys were mildly congested and slightly soft, particularly at the cortical region. The ureters were reddened. Two masses were observed in the reproductive tract. The first was a large, intramural and multinodular mass located at the cervico-uterine junction (Figure 4). The diameter was between 15 cm and 18 cm, weighing nearly 7 kg. This mass was seen to cause obstruction of the abdominal aorta and caudal vena cava. The second mass was located in the submucosa of the uterine body. It was spherical in shape, with the diameter of 4 cm and had a smooth surface but soft and caseous material on the interior. The uterus was thick-walled and filled with mucopurulent material and blood. In the endometrium of the left uterine horn, numerous cysts (diameter 3 mm to 2 cm) were observed. The ovaries were deprived of follicles.

**Figure 4.**
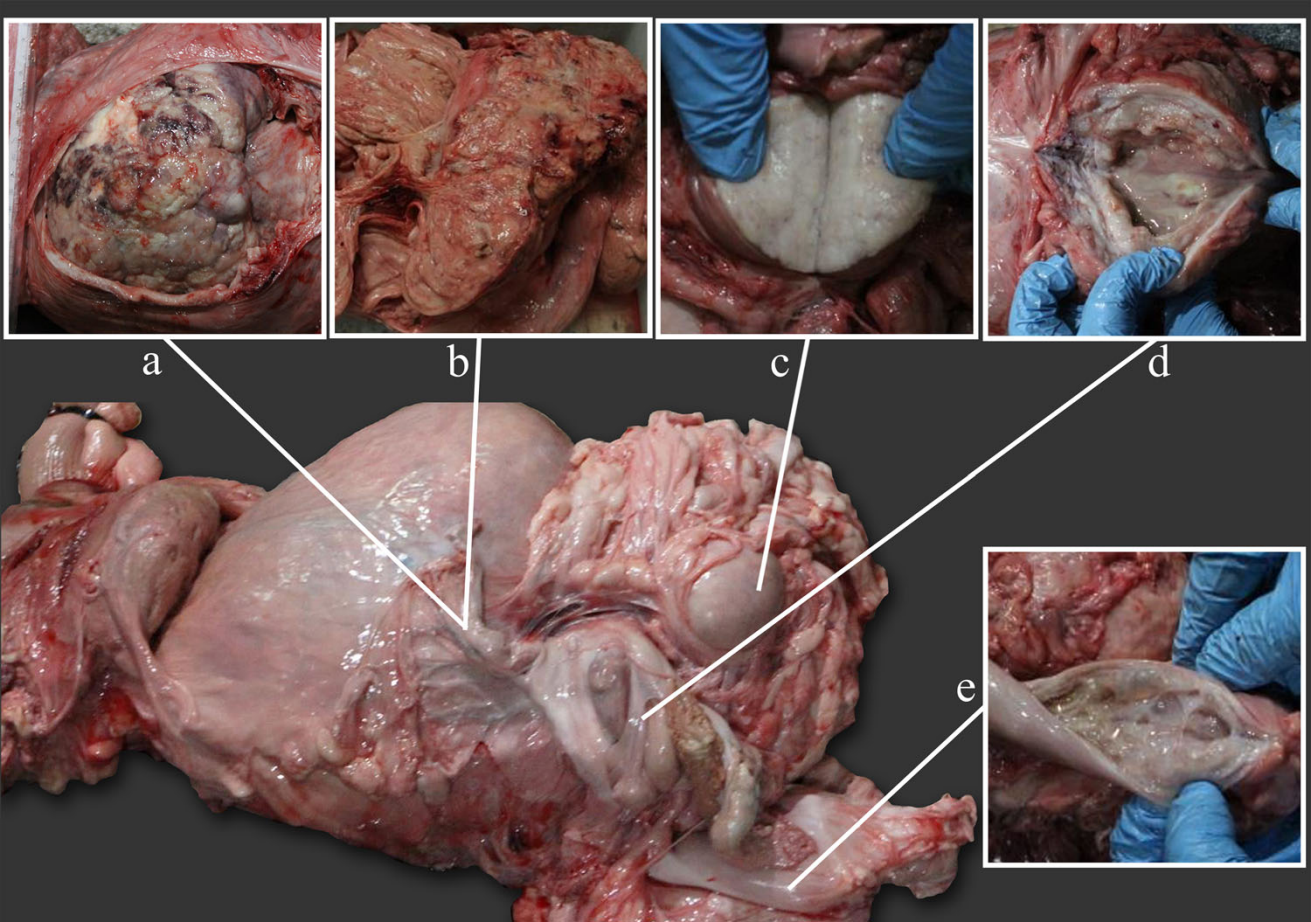
Gross lesions in the reproductive tract of a female Sumatran rhinoceros estimated to be between 20 and 25 years old. (a) Mass located at the cervico-uterine junction. (b) Cut surface of the cervico-uterine mass. (c) Cut surface of the mass located at uterine body. (d) Pyometra. (d) Cystic endometriosis in left uterine horn.

Samples of the above-mentioned organs and masses were collected, fixed in 10% neutral-buffered formalin, and routinely processed. The tissues were sectioned at 4 µm thick and stained with haematoxylin and eosin (HE). Samples of the masses were also examined via special staining using alcian blue (pH 2.5) and Masson’s trichrome, and immunohistochemistry (IHC) for immunodetection of alpha smooth muscle actin (SMA) (1:400; Dako, Petaling Jaya, Malaysia; Ref M0851), vimentin (1:600; Novusbio, Kuala Lumpur, Malaysia; Ref NB300-223), desmin (1:200; Novusbio, Ref NB120-15200), cytokeratin AE1/AE3 (ready-to-use; Dako, Ref M3515), epithelial membrane antigen (EMA) (ready-to-use; Dako), CD34 (1:100; Dako, M7156), and S100 (ready-to-use; Dako, Ref GA504).

Histological examination revealed that the liver was congested, with mild centrilobular necrosis. The spleen was markedly congested and hemorrhagic with moderate lymphoid depletion and hemosiderosis. The kidneys had severe interstitial nephritis with necrotic and desquamated tubular epithelium. Mild multifocal fibrosis was observed in the myocardium. In general, the two masses within the reproductive tract showed different histopathologic features, with the exception that both masses had mild vascular thrombosis. The cervico-uterine mass was well demarcated and diffusely composed of large, ovoid and polyhedral neoplastic mesenchymal cells. These cells exhibited intense eosinophilic cytoplasm and mild nuclear atypia. Occasionally, binucleation and chromatin clumping were observed (Figure 5a). The neoplastic cells were surrounded by wispy collagen fibers (Figure 5b) which were Alcian blue negative (Figure 5c). Extramedullary hematopoeisis (EMH) was evident in the tumour vascular lumen with the presence of immature cells, including the megakaryocytes (Figure 5d). Mitotic activity was low at 5/10 HPF. Immunohistochemistry on the neoplastic mesenchymal cells revealed intense intracytoplasmic staining for SMA and vimentin (Figure 5e and f), but the cells were tested negative for desmin, cytokeratin AE1/AE3, EMA, CD34, and S100.

**Figure 5.**
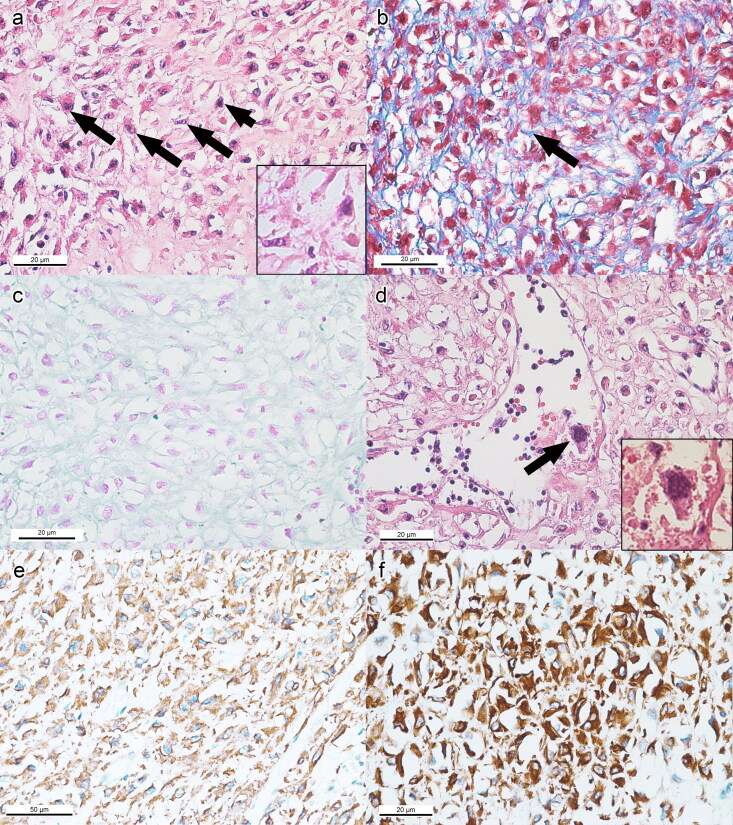
Routine histopathology, special stains, and IHC of the epithelioid leiomyoma of the cervico-uterine junction. (a) Diffused large, ovoid or polyhedral mesenchymal cells characterized by intense eosinophilic cytoplasm, binucleation (long arrows) and nuclear chromatin clumping (short arrow). X400, bar = 20 μm, HE. Inset: Higher magnification. (b) Neoplastic leiomyocytes surrounded by thin collagen fibers (arrow). X400, bar = 20 μm, Masson’s trichrome. (c) Negative staining of Alcian blue. X400, bar = 20 μm, Alcian blue. (d) Observation of megakaryocytes in the tumor vascular lumen. X400, bar = 20 μm, HE. Inset: Higher magnification. (e) Intracytoplasmic immunodetection of SMA. X200, bar = 50 μm, IHC. (f) Intense intracytoplasmic immunodetection of vimentin. X400, bar = 20 μm, IHC.

The smaller mass that was located at the uterine body was mainly composed of residual spindle cells with fibrillary eosinophilic cytoplasm (Figure 6a) and stained red with Masson’s trichrome (Figure 6b). The residual spindle cells were surrounded by glycosaminoglycan-rich matrix suggestive of myxoid degeneration (Figure 6c). Some areas showed densely packed mesenchymal cells exhibiting elongated cells with cigar-shaped nuclei. Mitotic activity was low at 2/10 HPF. The IHC examination showed strong intracytoplasmic positive staining for SMA, intracytoplasmic and occasionally intranuclear positive staining for vimentin, and partial intracytoplasmic positive staining for desmin (Figure 6d–f). Most areas with severe myxoid degeneration were negative for desmin. This uterine tumour was also negative for other tested markers.

**Figure 6.**
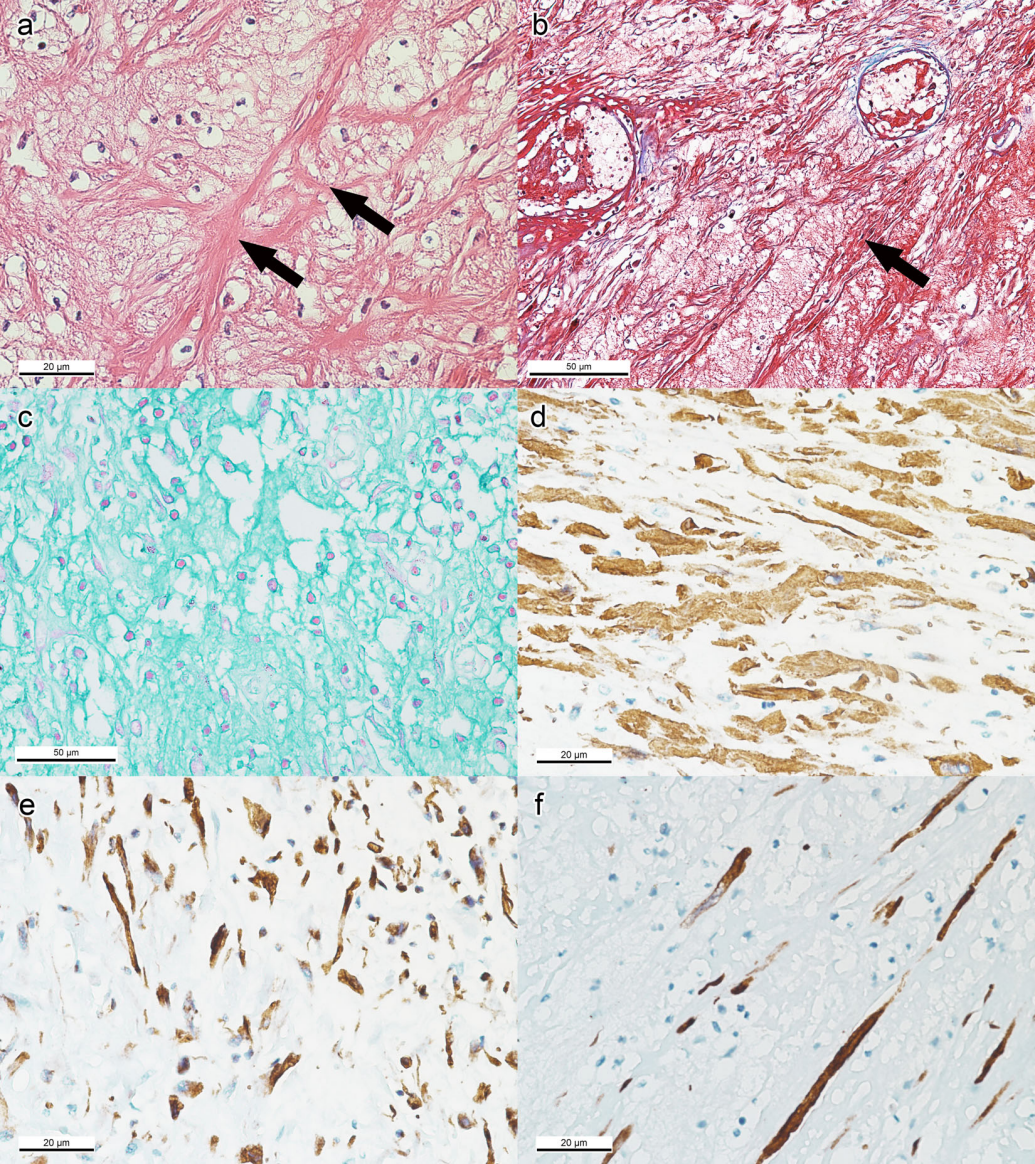
Routine histopathology, special stains, and IHC of myxoid leiomyoma of uterine body. (a) Residual mesenchymal cells with eosinophilic fibrillary cytoplasm (long arrows) separated by myxoid matrix. X400, bar = 20 μm, HE. (b) Fibrillar cytoplasm of neoplastic cells as confirmed by the red stain. X200, bar = 50 μm, Masson’s trichrome. (c) Presence of glycosaminoglycan-rich matrix suggestive of myxoid degeneration. X200, bar = 50 μm, Alcian blue. (d) Intracytoplasmic immunodetection of SMA in neoplastic cells. X400, bar = 20 μm, IHC. (e) Neoplastic cell with intracytoplasmic vimentin immunodetection. X400, bar = 20 μm, IHC. (f) Low distribution of desmin immunodetection. X400, bar = 20 μm, IHC.

Lesions in female reproductive tract of wildlife have been well-documented. Yet, the data were considered as limited, especially in critically endangered species. For megavertebrates like rhinoceros and elephants, among the possible contributing factors for development of asymmetric reproductive aging pathology could be their low abundance, habitat fragmentation, or being kept in captivity. These factors are closely associated with long stretches of non-reproductive periods (Hermes et al. 2004). Different types of female reproductive lesions in megavertebrates like Sumatran, white (*Ceratotherium simun simun*), black (*Diceros bicornis*), and greater one-horned (*Rhinoceros unicornis*) rhinoceroses, as well as Asian elephants (*Elephas maximus*) have been reported. These include uterine leiomyoma, cystic hyperplasia, chronic endometritis, uterine adenocarcinoma, fibroma, and vaginal hemangioma (Heidegger et al. 2016; Hermes et al. 2014; Hermes et al. 2004; Hermes et al. 2006; Rajeev et al. 2017; Reese et al. 1992; Schaffer et al. 2020; Schaffer et al. 1994; Wilson et al. 2010). The former two conditions accounted for up to 80% of the most common lesions in the reproductive tract of non-reproductive female rhinoceros and elephants (Hermes et al. 2004).

Gross, histopathology, and IHC examinations established the diagnoses of epithelioid leiomyoma of the cervico-uterine junction, and myxoid leiomyoma of the uterine body. Differential diagnoses of adenoma, carcinoma, and carcinosarcoma which could be ruled out based on histopathology and IHC, should be positive for cytokeratin and EMA (Rabban et al. 2019).

Because currently there is no validated subclassification of leiomyomas in veterinary medicine (Kennedy et al. 1998; Agnew and Maclachlan 2017; Schlafer and Foster 2016), the diagnoses were made based on the human medical nomenclature by World Health Organisation (WHO) (Arleo et al. 2015; Kurman et al. 2014). The current WHO classification of uterine leiomyoma are cellular leiomyoma, leiomyoma with bizarre nuclei (previously known as atypical leiomyoma), mitotically active leiomyoma, apoplectic leiomyoma, lipomatous leiomyoma (lipoleiomyoma), epithelioid leiomyoma, myxoid leiomyoma, dissecting leiomyoma, diffuse leiomyomatosis, intravenous leiomyomatosis, and metastasizing leiomyoma.

The histopathologic features of the cervico-uterine and uterine tumours seen in this case fulfil the hallmark criteria of epithelioid leiomyoma and myxoid leiomyoma as described in human medicine, respectively (Arleo et al. 2015; Mikami 2018). SMA is known to be a better screening protein for muscle tumours compared to desmin (Folpe and Cooper 2007). Both tumours in this case expressed SMA while immunodetection of desmin was only observed in the myxoid leiomyoma. The intensity of positive staining for both SMA and desmin negatively correlates with the grade of mesenchymal tumours (Turner and Goldsmith 2009). In addition, higher grade tumours have higher mitotic activity, and vice versa (Singer et al. 1996). This is in agreement with this case, where the myxoid leiomyoma has lower mitotic activity, and yielded partial positive immunodetection of desmin.

To our knowledge, no report has been made for variants of uterine leiomyoma in animals, with the exception of uterine lipoleiomyoma and angiolipoleiomyoma in dogs (Boisclair and Dore 2001; Percival et al. 2018). It is important to differentiate leiomyoma from leiomyoma variants and leiomyosarcoma, as they are associated with different degrees of aggressiveness in humans (Arleo et al. 2015). The association of different variants with different outcomes may as well become important in veterinary species. Diagnosis of these tumours requires histopathology and seldom of IHC (Arleo et al. 2015); however, obtaining samples in megavertebrates could be challenging. In this case, both tumour variants showed typical benign growth pattern. The leiomyoma at the cervico-uterine junction was eventually large enough to cause extramural compression of the abdominal aorta and caudal vena cava resulting in severe renal necrosis and failure. Observation of megakaryocytes suggests that extramedullary hematopoeisis has previously been described in uterine leiomyoma (Cui et al. 2014), possibly due to the chronic vaginal bleeding and/or paraneoplastic syndrome.

Clinical and/or pathological examinations of female Sumatran rhinoceroses (including this current case) that were captured in Malaysia and Indonesia between 1984 and 2018 have revealed that more than 90% had reproductive lesions, with uterus as the most commonly affected organ (Schaffer et al. 2020). Uterine tumours and cysts were reported as the most frequently observed reproductive tract abnormalities, with only one rhinoceros was noted with more than one uterine leiomyoma. However, no detailed pathology description of the mentioned case was published. In white rhinoceros, reproductive lesions are almost never encountered in wild population, but quite prevalent in the captive population (Hermes et al. 2014). Whereas in Sumatran rhinoceros, reproductive pathologies were noted in both wild and captive populations (Schaffer et al. 2020).

Other than clinical and pathological descriptions of the two leiomyoma variants, this report re-emphasizes the significance of reproductive diseases of Sumatran rhinoceros. Factors such as a critically small population, high risk of infertility, low success rate of captive breeding, lack of political support within and between countries, poaching, habitat loss and fragmentation (Zafir et al. 2011; Havmøller et al. 2016; Schaffer et al. 2020) may eventually lead to the extinction of Sumatran rhinoceros. More pregnancies are required not only for population growth, but also to prevent female rhinoceros reproductive lesions (Hermes et al. 2006). Sumatran rhinoceros in Malaysia had been declared extinct in the wild since March 2014 (Havmøller et al. 2016), and this rhinoceros was the last of its species in Malaysia. The death of the last Malaysia’s Sumatran rhinoceros should be viewed as a serious issue by other countries, their policy makers, and worldwide experts so they will work hand-in-hand to avoid the extinction of this species.
